# Retraction: The Complex Regulation of Tanshinone IIA in Rats with Hypertension-Induced Left Ventricular Hypertrophy

**DOI:** 10.1371/journal.pone.0300689

**Published:** 2024-03-12

**Authors:** The PLOS ONE Editors

After this article [1] was published, concerns were raised about Figure 2 and Figure 4.

Specifically:

- In Figure 2A, the lower left region of the Tan (10) panel appears similar to the upper right region of the Tan (15) panel.
- In Figure 2D, lane 1 of the GAPDH panel appears similar to lane 5 of the GAPDH panel of Figure 2E.
- In Figure 4A, the upper right region of the top 5% GS panel appears similar to the lower left region of the top Tan (15) panel.
- In Figure 4A, the lower right region of the bottom 5% GS panel appears similar to the upper left region of the bottom Tan (5) panel.

The authors did not respond to queries about these concerns. In the absence of underlying data, the above issues remain unresolved.

In light of the above concerns, which call into question the reliability of these data, the *PLOS ONE* Editors retract this article.

All authors either did not respond directly or could not be reached.

## References

[1] Pang H, Han B, Yu T, Peng Z (2014) The Complex Regulation of Tanshinone IIA in Rats with Hypertension-Induced Left Ventricular Hypertrophy. PLoS ONE 9(3): e92216. doi: 10.1371/journal.pone.0092216 24647357 PMC3960224

